# Prediction of Coronary Artery Aneurysms in Children With Kawasaki Disease Before Starting Initial Treatment

**DOI:** 10.3389/fped.2021.748467

**Published:** 2021-09-30

**Authors:** Ching-Ying Huang, Nan-Chang Chiu, Fu-Yuan Huang, Yen-Chun Chao, Hsin Chi

**Affiliations:** ^1^Department of Pediatrics, MacKay Children's Hospital, Taipei City, Taiwan; ^2^Department of Pediatrics, MacKay Memorial Hospital, Taipei City, Taiwan; ^3^Department of Medicine, MacKay Medical College, New Taipei City, Taiwan; ^4^MacKay Junior College of Medicine, Nursing and Management, Taipei City, Taiwan

**Keywords:** coronary artery aneurysm, Kawasaki disease (KD), coronary z-score, prediction, outcome

## Abstract

**Background:** Precisely predicting coronary artery aneurysms (CAAs) remains a clinical challenge. We aimed to investigate whether coronary dimensions adjusted for body surface area (Z scores) on baseline echocardiography and clinical variables before primary treatment could predict the presence of late CAAs.

**Methods:** We conducted a retrospective study including children hospitalized for Kawasaki disease and received intravenous immunoglobulin within 10 days of illness. We defined late CAAs as a maximum Z score (Zmax) ≥2.5 of the left main, right, or left anterior descending coronary artery at 11–60 days of illness. Associations between late CAAs and clinical parameters and baseline maximum Z scores were analyzed.

**Results:** Among the 314 included children, 31 (9.9%) had late CAAs. Male, higher C-reactive protein, and higher baseline Zmax were risk factors of late CAAs. Late CAAs were significantly associated with baseline Zmax ≥2.0 vs. <2.0 (25 [32.5%] vs. 6 [2.5%], *P* < 0.001). The odds ratio for late CAAs among the patients with baseline Zmax ≥2.0 vs. <2.0 was 18.5 (95% confidence interval, 7.23 to 47.41, *P* < 0.001). The sensitivity, specificity, positive predictive value, and negative predictive value of baseline Zmax ≥2.0 for the presence of later CAAs were 80.6, 81.6, 32.5, and 97.5%, respectively.

**Conclusions:** Findings from this study suggest that Zmax ≥2.0 of coronary arteries on baseline echocardiography may be used to predict children at a high risk of late CAAs and allow for targeted early intensification therapy.

## Introduction

Kawasaki disease (KD) is an acute vasculitis that mainly affects infants and young children, with Taiwan having the third-highest incidence (69 in 1,00,000 children <5 years of age) worldwide (1). The development of coronary artery aneurysms (CAAs) is the most significant complication of KD and is associated with higher risks of impairment of ventricular mechanics, thrombosis, stenosis, unstable angina, myocardial infarction and even death (2–7). Early recognition of KD and treatment with intravenous immunoglobulin (IVIG) in conjunction with aspirin have been shown to reduce the risk of coronary artery formation; nonetheless, despite the intervention, CAAs still occur in 2 to 6% of patients in the subacute and convalescent stages (8–10). Adjuvant anti-inflammatory therapy, administered together with primary standard IVIG treatment in the acute stage, has been shown to reduce the progression of coronary dilation (11, 12). However, the time window in which to diagnose and treat KD with intensification is brief, as the primary therapy should be started within the first 10 days of illness to prevent long-term complications (13, 14). Therefore, a reliable method of promptly identifying children at a high risk of later CAAs is needed to tailor treatment (15).

Risk factors for CAAs include male sex (16), IVIG treatment beyond 10 days of symptom onset (16, 17), increased C-reactive protein (CRP) (16), extreme age (18, 19), tachycardia (20), and abnormal initial echocardiography (21). Of these, coronary artery size at diagnosis seems to be most related to the progression or persistence of CAAs (22). A higher initial coronary dimension adjusted for body surface area (Z-score) has been associated with the development of CAAs within the first six weeks of disease, with each one-unit increase in z-score at diagnosis increasing the risk of major adverse cardiac events by 10% (21, 23). In this study, we aimed to identify predictors for CAAs and to determine whether coronary Z scores on baseline echocardiography are associated with the presence of CAAs.

## Methods

### Population

We retrospectively reviewed charts of all children (aged 0–18 years) diagnosed with KD and administered IVIG (2 g/kg as a single infusion) at MacKay Children's Hospital from January 1, 2012, through December 31, 2018. The exclusion criteria were: (1) not the first episode of KD (recurrent KD); (2) no or unknown IVIG treatment; (3) delayed IVIG treatment (>10 days after fever onset); (4) no baseline echocardiography data (either before or within 2 days of first IVIG administration); (5) no available follow-up echocardiography data during 11–60 days after illness onset; (6) coexisting congenital heart disease (except for bicommissural aortic valve without stenosis or regurgitation); (7) first laboratory studies obtained ≥1 day after IVIG administration; (8) insufficient laboratory data to calculate established risk scores. This study was approved by the MacKay Memorial Hospital Institutional Review Board (18MMHIS183e).

### Data Collection and Definitions

Data on demographic characteristics, presenting symptoms, physical examination findings, laboratory values, echocardiographic data, and treatment were recorded. Patients were classified as complete KD if they had fever plus the presence of ≥4 of the 5 principal clinical features; those who had fever plus <4 clinical criteria with compatible laboratory or echocardiographic findings were classified as incomplete (atypical) KD as per the American Heart Association (AHA) definition (24). The day of illness onset was defined as the first day of fever. IVIG resistance was defined as persistent or recrudescent fever at least 36 h and <7 days after the end of the first IVIG infusion. Coronary artery internal diameters in the left main coronary artery (LMCA), left anterior descending artery (LAD), and right coronary artery (RCA) obtained by echocardiography were converted to Z scores using a model derived from body surface area-adjusted Z scores based on data from healthy Taiwanese children (25). Z scores were calculated at baseline and for studies obtained 11–60 days after illness onset. The maximum Z score (Zmax) was defined as the largest Z score of the LMCA, LAD, or RCA on echocardiography. Patients were defined as having late CAAs if they had a Z score ≥2.5 in the LMCA, LAD, or RCA at 11–60 days after illness onset. Small, medium, and giant (or large) aneurysms were classified according to 2017 American Heart Association guidelines as Z scores of ≥2.5 to <5, ≥5 to <10 (and absolute internal diameter <8 mm), and ≥10 (or ≥8 mm), respectively. We collected the echocardiographic data of children at baseline, at 11–60 days, and 2–12 months following fever onset. The subsequent echocardiograms were assessed for persistence or regression of the CAAs, and the frequency varied depending on the severity of coronary artery lesions. For those who developed CAAs, the follow-ups were assessed until regression of aneurysms were recorded.

### Statistical Analysis

Descriptive statistics were used for demographic, clinical, and echocardiographic data. Continuous variables were described as mean (standard deviation) and categorical variables as numbers and percentages. Data were compared using χ^2^ or Fisher's exact test for categorical variables and Student *t* or Mann-Whitney test for continuous variables. Crude odds ratios for late CAAs among children with clinical and laboratory variables were estimated using univariate analysis. Risk factors identified by univariate analysis were applied for multivariate analysis, the logistic regression with a stepwise regression procedure. A receiver operating characteristic (ROC) curve was generated to identify the predictive validity of each parameter that was found to be significant by multivariate analysis. Youden J statistics were used to determine the most discriminating cutoff value. Sensitivity, specificity, and predictive values of the targeted parameter for late CAAs were calculated. Proportions of children with late CAAs were further categorized into five groups according to baseline Zmax as follows: <1.5, ≥1.5 to <2.0, ≥2.0 to <2.5, ≥2.5 to <3.0, and ≥3.0. A quadratic model was used to estimate the rate of late CAAs in each category based on the baseline Z scores. All *P* values were based on 2-sided tests and were considered statistically significant at <0.05. All statistical analyses were conducted using SPSS version 21.0 (IBM, Armonk, New York).

## Results

A total of 314 patients with KD were enrolled, of whom 178 (56.7%) were male. Overall, 109 (34.7%) patients received a diagnosis before 1 year of age, 197 (62.7%) between 1 and 5 years of age, and 8 (2.5%) at 5 years of age or older. Thirty-nine (12.4%) patients were classified as having incomplete (atypical) KD. Thirty (9.5%) of incomplete (atypical) KD patients had three clinical criteria, and night (2.9%) had less than two clinical criteria for KD. Patients with incomplete (atypical) KD were younger than those with complete KD (median [IQR]: 10.2 [6.7–17.5] months vs. 18.2 [10.7–29.7] months, *P* < 0.001). The median time between the onset of fever and IVIG administration was 6 days (IQR: 5–7 days). Twenty-one patients (6.7%) did not respond to the first dose of IVIG. Among the patients with IVIG resistance, 15 (4.8%) received the second dose of IVIG, 2 (0.6%) received corticosteroids as the second-line treatment, and 3 (1%) received a combination of a second dose of IVIG and corticosteroids.

Overall, 31 patients (9.9%) had the presence of CAAs at 11–60 days of illness. Patients with or without the presence of late CAAs had no differences in days from illness onset to IVIG treatment (median [IQR]: 6 (5–8) vs. 6 (5–7), *P* = 0.24) and doses (mg/kg/day) of acetylsalicylic acid administration (median [IQR]: 57.1 [44.4–80.9] vs. 50.7 [37.6–80.0], *P* = 0.17), proportions of combination primary therapy (0 vs. 1.8%, *P* = 1.0), and days of baseline echocardiograms (median [IQR]: 6 (5–8) vs. 6 (5–7), *P* = 0.74). The median time from the initial treatment to second/third echocardiography was 15 (IQR: 11–22) days/50 (IQR: 42–80) days.

The clinical variables identified by univariate analysis to be significant predictors of late CAAs were as follows: male gender, CRP, maximum Z score on baseline echocardiography, and IVIG resistance (Table 1). Patients with IVIG resistance had a higher rate of late CAAs compared with those with IVIG response (5 [16.1%] vs. 16 [5.7%], *P* = 0.04). The Zmax of IVIG non-response patients ranged from 2.9 to 6.1 at 11–60 days after illness onset. All the five IVIG-resistant cases with late CAAs received a second dose of IVIG, and the two received additional corticosteroids as the rescue therapy had Zmax < 3.0. We did not put IVIG resistance into multivariate analysis because it cannot be recognized before primary treatment.

**Table 1:** Comparisons between patients with and without coronary artery aneurysms at 11 to 60 days of illness.

	**Case No**.	**With CAAs (*N* = 31)**	**Without CAAs (*N* = 283)**	**Univariate analysis**	
				**OR (95%)**	***P***
Age at illness onset in months	314			1.01 (0.98–1.03)	0.66
Mean (SD)		22.1 (16.4)	16.9 (8.0–33.2)		
Median (IQR)		20.8 (15.8)	17.4 (9.9–26.9)		
< 1 Year		11 (35.5)	98 (34.6)	1.038 (0.478–2.255)	0.92
1–5 Year		20 (64.5)	177 (62.5)	1.089 (0.502-2.361)	0.83
>5 Year		0 (0)	8 (2.8)	—	—
Male sex	314	25 (80.6)	153 (54.1)	3.54 (1.41–8.89)	**0.007**
Incomplete KD	314	7 (22.6)	32 (11.3)	2.29 (0.91–5.73)	0.08
WBC, x10^9^ cells/L	313			1.0 (1.0–1.0)	0.61
Mean (SD)		15.1 (5.7)	14.5 (5.8)		
Median (IQR)		13.8 (10.8–17.7)	13.8 (10.7–17.7)		
% Neutrophile	314			1.02 (0.99–1.05)	0.11
Mean (SD)		64.2 (12.0)	59.9 (14.4)		
Median (IQR)		61.0 (56.0–74.0)	61.5 (50.5-70.4)		
Hemoglobin, g/dL	311			0.93 (0.66–1.31)	0.66
Mean (SD)		10.9 (1.1)	11.1 (1.0)		
Median (IQR)		11.0 (10.4–12.0)	11.1 (10.4–11.8)		
Platelet count, x10^9^/L	310			1.0 (.99–1.0)	0.47
Mean (SD)		355.1 (108)	328.8 (118.1)		
Median (IQR)		329 (275.3–422)	326.5 (274–386.7)		
CRP, mg/dL	257			1.06 (1.0–1.12)	**0.04**
Mean (SD)		11.1 (6.6)	8.3 (6.4)		
Median (IQR)		8.9 (6.4–15.1)	6.5 (3.6–11.4)		
Sodium, mmol/L	190			0.98 (0.83–1.14)	0.75
Mean (SD)		135.2 (3.8)	135.5 (2.9)		
Median (IQR)		135.0 (132.5–139)	135.5 (134–137)		
Albumin, g/dL	214			0.48 (0.21–1.12)	0.09
Mean (SD)		3.7 (0.5)	3.9 (0.5)		
Median (IQR)		3.8 (3.4–4.1)	4.0 (3.6–4.3)		
AST, IU/L	276			1.0 (0.99–1.0)	0.83
Mean (SD)		84 (99)	79 (119)		
Median (IQR)		44 (31–83)	42 (29–73)		
ALT, IU/L	276			1.0 (0.99–1.0)	0.90
Mean (SD)		82 (95)	84 (99)		
Median (IQR)		36 (15–124)	34 (19–104)		
Maximum Z score on baseline echocardiography	314			5.51 (3.26–9.31)	** < .001**
Mean (SD)		2.7 (0.9)	1.4 (0.8)		
Median (IQR)		2.8 (2.2–3.4)	1.4 (0.9–1.8)		
IVIG resistance	314	5 (16.1)	16 (5.7)	3.21 (1.09–9.47)	**0.04**

*Data are presented as N (%) or mean (SD), or median (IQR)*.

*ALT, aspartate aminotransferase; AST, alanine aminotransferase; CAAs, coronary artery aneurysms; CRP, C-reactive protein; KD, Kawasaki disease; IQR, interquartile range; IVIG, intravenous immunoglobulin; SD, standard deviation; WBC, white blood cell count: —value cannot be calculated because of the limited number of cases*.

*The bold values mean that the results were statistically significant*.

The other three variables were included in the multivariate logistic regression analysis with the following results: odds ratio (OR) = 2.33 (95% CI: 0.76–7.13, *P* = 0.14) for male gender, OR = 1.04 (95% CI: 0.76–7.13, *P* = 0.24) for CRP, and OR = 4.54 (95% CI: 2.53–8.17, *P* < 0.001) for baseline maximum Z score. Baseline maximum Z score was identified as the sole significant variable at the time of diagnosis that distinguished those who would have the presence of late CAAs from those who would not have at 11–60 days of illness. ROC curve was performed to determine the baseline Zmax that may predict the presence of late CAAs. The cutoff value of baseline Zmax assessed by Youden *J* statistics was 1.96. We selected a baseline Z score of ≥2.0 as the cutoff value to predict late CAAs. Baseline Zmax ≥2.0 was significantly associated with late CAAs compared with baseline Zmax <2.0 (25 [32.5%] vs. 6 [2.5%], *P* < 0.001).

Table 2 presents the predictive ability of baseline Zmax ≥2.0 for the development of late CAAs in our cohort. The sensitivity, specificity, positive predictive value, and negative predictive value were 80.6, 81.6, 32.5, and 97.5%, respectively. Compared with the patients with a Zmax < 2.0, those with baseline Zmax ≥ 2.0 had a much higher risk of CAAs at 11–60 days after illness onset (OR: 18.5; 95% CI: 7.23 to 47.41; *P* < 0.001).

**Table 2:** Maximum Z score ≥2.0 on baseline echocardiography as a predictor of late coronary artery aneurysms at 11 to 60 days of illness.

**Measure**	**Value (95% CI)**	***P***
Sensitivity	0.81 (0.63–0.91)	
Specificity	0.82 (0.76–0.85)	
Positive predictive value	0.32 (0.23–0.44)	
Negative predictive value	0.97 (0.95–0.99)	
False positive rate	0.18 (0.14–0.23)	
Accuracy	0.82 (0.77–0.86)	
Positive likelihood ratio	4.39 (3.25–5.92)	
Negative likelihood ratio	0.24 (0.12–0.49)	
AUC of ROC	0.86 (0.78–0.98)	< .001

*AUC, area under the curve; ROC, receiver operating characteristic*.

**Figure 1:**
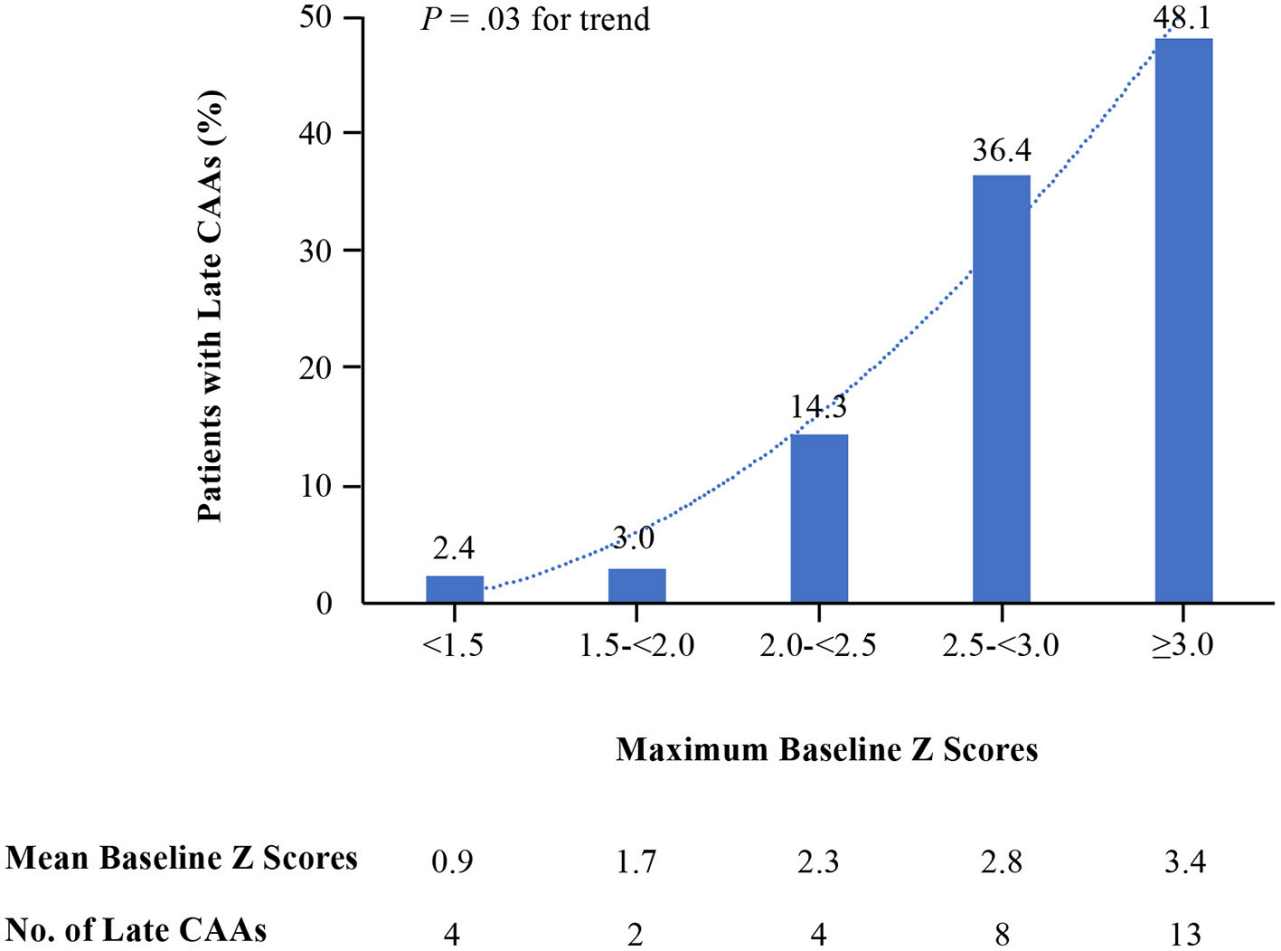
Baseline maximum Z scores and rates of late coronary artery aneurysms.

In baseline echocardiography, 170 (54.1%), 67 (21.3%), 28 (8.9%), 22 (7.0%) and 27 (8.6%) patients had a baseline Zmax of <1.5, 1.5 to <2.0, 2.0 to <2.5, 2.5 to <3.0, and ≥3.0, respectively. The rates of late CAAs in each group, based on maximum baseline Z scores, were revealed in Figure 1. Proportions of patients with late CAAs for those with baseline Zmax < 1.5 and 1.5 to <2.0 were low (2.4 and 3.0%, respectively) and increased to 14.3% with baseline Zmax 2.0 to <2.5. Overall, the trend of rate of late CAAs significantly increased with each higher cutoff value of baseline Zmax (*P* = 0.03) at 11–60 days after illness onset. Amongst the 77 patients with baseline Zmax ≥2.0, 34 were <1 year and 43 were between 1–5 years. For patients with baseline Zmax ≥2.0, there was no statistical difference between the two age groups for rates of late CAAs (<1 year: 29.4% vs. 1–5 years: 34.8%, *P* = 0.61).

Comparisons of late CAAs between the patients with baseline Zmax <2.0 and ≥2.0 are summarized in Table 3. The average Zmax of late CAAs was higher in the group with baseline Zmax ≥2.0 (3.2 ± 0.6 vs. 3.6 ± 1.5; *P* = 0.99). All cases with baseline Zmax <2.0 who later had CAAs developed small aneurysms at 11–60 days after illness onset. In the group with baseline Zmax ≥2.0, two cases developed medium aneurysms, and one had a giant aneurysm with a dimension of 10.1 mm in the RCA.

**Table 3:** Comparisons of children with late coronary artery aneurysms by maximum coronary Z score on baseline echocardiography.

	**Baseline Zmax**	
	** <2.0 (*N* = 237)**	**≥2.0(*N* = 77)**
Late CAA, N (%)	6 (2.5%)	25 (32.5%)
Zmax of late CAA		
Mean (SD)	3.2 (0.6)	3.6 (1.5)
Median (IQR)	3.1 (2.9–3.7)	3.2 (2.7–3.7)
Type of late CAA, N		
Small	6	22
Medium	0	2
Giant	0	1

*CAA, coronary artery aneurysm; Zmax, maximum Z score*.

Among the 31 patients with late CAAs at 11–60 days of illness onset, 27 had their coronary arteries regress to normal Z scores 2–12 months after illness onset, and two regressed in the 1–2 years follow-up period. Two patients (0.6%) had aneurysms that persisted until the last follow-up (30 and 33 months after illness onset, respectively). For the patients who recovered from late CAAs, those with baseline Zmax ≥2.0 took a longer average time to return to a normal coronary artery dimension on echocardiography than those with baseline Zmax <2.0 (3.4 ± 2.3 vs. 2.4 ± 1.5 months; *P* = 0.39).

## Discussion

In this analysis of echocardiography using body surface area-adjusted coronary artery dimensions in children with KD, the rate of late CAAs increased with each higher category of baseline maximum Z score. The agreement between baseline Zmax ≥2.0 on baseline echocardiography and the presence of late CAAs at 11–60 days of illness was strong, as Zmax ≥2.0 on baseline echocardiography increased the risk of CAAs at 11–60 days of illness by a factor of 18.5. More than four-fifths of the children who had CAAs at 11–60 days of illness were identified in baseline echocardiography, suggesting that these children at risk of developing CAAs may have been identified early and received tailored primary treatment using this approach.

As reported in previous studies, the patients who developed coronary aneurysms had higher maximum Z scores from the initial assessment. Liu et al. reported that a maximal Z score ≥2.5 during the acute KD phase predicted persistent or progressive coronary aneurysms at one month after KD onset in more than two-thirds of their patients (26). In addition, McCrindle et al. reported that if the Zmax was ≥2.5 in initial echocardiography, the Z score remained ≥2.5 over 5 weeks of follow-up in 78% of their subjects, whereas only 6% with an initial maximum Z score <2.5 increased above 2.5 (27). However, 1 in 7 patients in our cohort with baseline Z score ≥2.0 and <2.5 (i.e., coronary artery dilatation) developed late CAAs during the subacute and convalescent stages, among whom 1 developed a giant CAA. Using baseline Z score ≥2.5 instead of ≥2.0 as a predictor for later CAAs may therefore miss some serious cases.

One-third of our patients with baseline Zmax ≥2.0 of the LMCA, RCA, or LAD developed CAAs during 11–60 days of illness. A recent study in a North American center showed that baseline Zmax ≥2.0 of the RCA or LAD was associated with later CAAs at 4–8 weeks after illness onset (15). On the other hand, Ma et al. observed peak coronary artery dilation at around day 11 from the onset of fever in patients with KD and stressed the importance of repeat echocardiograms during the second week of illness (28). Histologically, inflammation in the intima and adventitia has been shown to merge by day 10 of illness, with coronary artery dilatation occurring within the following 2 days (29). These reports provide evidence that it is necessary to monitor for CAAs in the subacute phase (usually 10 days to 4 weeks after the onset of illness) in patients with KD. The guideline of AHA recommends repeating echocardiography within 1 to 2 weeks after treatment for uncomplicated patients (24). Based on our findings, a closer follow-up within one week after initial treatment may be considered for patients with coronary artery dilatation on the initial echocardiography.

Z scores of the LMCA need to be interpreted with caution due to the more common anatomic variations in this artery (30). It can be argued that our findings may have overestimated the incidence of baseline coronary artery abnormalities due to the theoretical possibility of non-specific coronary artery dilatation caused by localization to the more variable LMCA or only mild dilatations (i.e., Z score only slightly above 2.0). Our data, however, suggest that this explanation is less likely. In our cohort, 28 (36%) patients had a baseline Z score ≥2.0 isolated to the LMCA, and most of these patients (71%) had Z scores ≥2.5, meaning a significant abnormality of the coronary artery. Our findings are also supported by a previous study in which the authors concluded that patients with KD with coronary artery diameter variations in the LMCA and RCA were at an increased risk of treatment failure and that those with increased systemic inflammatory parameters may be at a higher risk of coronary artery events (31). Therefore, we calculated LMCA, RCA, and LAD Z scores and targeted the coronary lesions at 11–60 days after illness onset to maximize the detection of late CAAs.

Sensitivity and specificity of baseline Zmax were good for the prediction of the later development of CAAs in our patients. However, it had a low positive predictive value, which may be due to the regression of coronary arteries from a higher to normal Z score after IVIG treatment. Conversely, the negative predictive value was 97 %, as patients with baseline Zmax <2.0 were less likely to develop CAAs at 11–60 days after illness onset.

Most children with KD have good clinical outcomes after treatment with a single dose of IVIG in the first 10 days of illness; however, around 10% of children with KD still had CAAs in the first 60 days of illness in our study. The children with a baseline Zmax ≥ 2.0 developed larger CAAs in the subacute and convalescent stages. Their affected coronary arteries took more time to return to normal dimensions compared to the children with a baseline Zmax < 2.0, although the difference was not statistically significant. Although most CAAs may remodel to a normal lumen diameter over time, the appearance of aneurysm regression on echocardiography can be a result of myofibroblast cell proliferation or layered thrombus that may be followed by persistent abnormal vascular wall morphology and vascular dysfunction at the site of the regressed coronary aneurysm (32, 33). Moreover, coronary flow reserve has been shown to be abnormal in patients with a history of transiently dilated coronary arteries, and inflammatory changes in coronary arteries have been shown to exist even in the absence of aneurysms (34, 35). A significant association between the maximal Z score of the coronary arterial internal diameter and a higher risk of later coronary events in patients with a history of KD has also been reported (2, 23, 36). In order to reduce coronary arterial wall damage, high-risk children should be identified early at the time of diagnosis to allow for effective primary treatment.

This study has several limitations. First, the study is limited by its retrospective design. Considering that late CAAs may spontaneously regress over time, a prospective study with more frequently repeated echocardiograms may better detect the occurrence of late CAAs. We may have missed some less severe cases because follow-up echocardiography for patients without baseline CAAs tends to be postponed, and only patients with outcome data from echocardiograms during the study period were included. In addition, most of the patients with IVIG resistance received additional therapy, which would have biased our study towards less positive results by improving coronary artery outcomes at 11–60 days of illness. Moreover, the relatively small number of patients with late CAAs restricted our ability to reach some comparative conclusions between those with baseline Zmax < 2.0 and ≥2.0.

## Conclusion

Our findings demonstrate that the maximum Z score of coronary arteries on baseline echocardiography may be a predictor of the presence of CAAs at 11–60 days of illness. Z score of coronary arteries may therefore be an imaging biomarker to promptly identify patients at risk of developing CAAs to allow for tailored primary therapy to prevent the development and progression of CAAs. Further studies are needed to validate our findings in other populations.

## Data Availability Statement

The raw data supporting the conclusions of this article will be made available by the authors, without undue reservation.

## Author Contributions

C-YH and HC conceptualized and design the study. C-YH wrote the first draft of the manuscript. N-CC, F-YH, and Y-CC collected and organized the database. All authors critically reviewed, revised the manuscript, and agreed to the published version of the manuscript. All authors contributed to the article and approved the submitted version.

## Conflict of Interest

The authors declare that the research was conducted in the absence of any commercial or financial relationships that could be construed as a potential conflict of interest.

## Publisher's Note

All claims expressed in this article are solely those of the authors and do not necessarily represent those of their affiliated organizations, or those of the publisher, the editors and the reviewers. Any product that may be evaluated in this article, or claim that may be made by its manufacturer, is not guaranteed or endorsed by the publisher.
